# 5,7-Dimethoxyflavone Attenuates Obesity-Associated Muscle Atrophy via AMPK and PI3K/Akt Pathways in *ob/ob* mice

**DOI:** 10.4014/jmb.2604.04003

**Published:** 2026-05-28

**Authors:** Mi-Bo Kim, Sunkyu Lee, Chae Young Moon, Jae-Kwan Hwang, Hyunju Kang

**Affiliations:** 1 Department of Food Science and Nutrition, Pukyong National University, Busan 48513, Republic of Korea; 2 Department of Biotechnology, College of Life Science and Biotechnology, Yonsei University, Seoul 03722, Republic of Korea; 3 Department of Food and Nutrition, Keimyung University, Daegu 42601, Republic of Korea; 4 Graduate Program in Bioindustrial Engineering, College of Life Science and Biotechnology, Yonsei University, Seoul 03722, Republic of Korea; 5 School of Food Science and Biotechnology, Kyungpook National University, Daegu 41566, Republic of Korea; 6 Food and Bio-Industry Research Institute, Kyungpook National University, Daegu 41566, Republic of Korea

**Keywords:** 5,7-dimethoxyflavone, Anti-obesity, Muscle atrophy, AMPK, PI3K/Akt pathways, Muscle function

## Abstract

5,7-Dimethoxyflavone (DMF), a bioactive flavone derived from *Kaempferia parviflora* Wall. ex Baker, has been reported to exhibit anti-obesity and muscle-protective potential; however, its effects on obesity-related muscle dysfunction and the underlying mechanisms in a leptin-deficient model remain to be fully elucidated. This study investigated the dual effects of DMF, on obesity and muscle atrophy in *ob/ob* mice. Male *ob/ob* mice were randomly assigned to vehicle- or DMF (20 or 50 mg/kg/day)–treated groups and orally administered DMF for 8 weeks, with age-matched wild-type mice serving as controls. Adiposity, skeletal muscle mass, grip strength, treadmill exercise endurance, and related molecular signaling pathways were evaluated. DMF significantly decreased body weight, fat volume, and fat mass without affecting appetite, suggesting a potential shift in energy partitioning. Mechanistically, DMF was associated with reduced expression of adipogenic transcription factors and lipogenic enzymes by upregulating AMP-activated protein kinase in epididymal fat. Conversely, DMF markedly increased muscle fiber size and muscle mass, which resulted in enhanced muscle function, such as exercise endurance and grip strength. At the molecular level, DMF activated the phosphatidylinositol 3-kinase / protein kinase B pathway, subsequently stimulating the mammalian target of rapamycin pathway and the concomitant phosphorylation of forkhead box O3a. Mitochondrial biogenesis-related markers were also upregulated by DMF. Overall, DMF may serve as a potential functional agent to mitigate obesity-induced muscle loss by modulating metabolic and atrophic signaling pathways.

## Introduction

Obesity-induced muscle atrophy is a metabolic disorder characterized by both progressive loss of skeletal muscle mass and function, along with excessive adipose tissue accumulation [1, 2]. This excessive adiposity is strongly linked to major metabolic disturbances, including insulin resistance, dyslipidemia, and type 2 diabetes, and its concurrence with sarcopenia markedly accelerates skeletal muscle deterioration. Supporting this pathological interaction, genetically obese mouse models deficient in either leptin or its receptor exhibit significantly reduced muscle mass compared with their wild-type counterparts [3]. Similarly, both quantitative and qualitative declines in skeletal muscle mass with increased adiposity have been observed in obese individuals [4, 5], suggesting that excess adipose tissue and its secretions have an adverse effect on skeletal muscle. Thus, effective strategies for managing obesity-related metabolic disorders must not only reduce fat accumulation but also preserve or enhance skeletal muscle mass and functional capacity [6, 7]. Identifying a single therapeutic agent capable of simultaneously modulating adiposity and muscle atrophy remains a critical research objective.

Obesity-induced muscle atrophy develops through the intertwined dysregulation of adipose tissue and skeletal muscle metabolism, implicating central molecular pathways that govern energy balance and protein turnover. Among these, AMP-activated protein kinase (AMPK) is a master regulator of cellular energy homeostasis [8]. Its activation suppresses adipogenesis and promotes energy expenditure, making it a key target for anti-obesity strategies [9]. Concurrently, the phosphatidylinositol 3-kinase (PI3K)/protein kinase B (Akt) pathway is widely recognized as the primary signaling cascade that promotes skeletal muscle protein synthesis and inhibits atrophy [10]. Therefore, therapeutic approaches capable of concurrently activating the AMPK axis in adipose tissue and enhancing PI3K/Akt-mediated anabolic signaling in skeletal muscle may offer a compelling strategy for correcting the dual metabolic impairments associated with obesity-induced muscle atrophy.

5,7-dimethoxyflavone (DMF), a major constituent of *Kaempferia parviflora* Wall. ex Baker (black ginger), exhibits multiple beneficial biological activities, including anti-obesity, anti-inflammatory, and muscle-protective effects [11-13]. Our previous studies further support these functions by demonstrating that DMF suppresses adipogenesis and fat accumulation in high-fat diet-induced obese mice and enhances muscle mass and performance in aged mice with sarcopenia [12, 14, 15]. However, although these findings highlight the metabolic and muscle-protective potential of DMF, the high-fat-diet model does not fully recapitulate the simultaneous presence of severe adiposity and intrinsic muscle deficits characteristic of obesity-induced muscle atrophy. Consequently, the effect of DMF on obesity-induced muscle atrophy has not been evaluated in *ob/ob* mice, a genetic model that inherently exhibits both obesity-associated metabolic dysfunction and baseline muscle wasting. Notably, the present study demonstrates for the first time that DMF is associated with attenuation of obesity and mitigation of muscle atrophy in *ob/ob* mice, potentially involving modulation of AMPK signaling and the PI3K/Akt pathway, supporting a possible link to the core metabolic defects of obesity-associated muscle atrophy.

## Materials and Methods

### Isolation of DMF from *K. parviflora*

Dried rhizomes of *K. parviflora* were collected from Bangkok, Thailand. The dried rhizomes of *K. parviflora* were ground and extracted with 95% ethanol for 3 h at 60°C. The resulting extract (KPE) was obtained by filtration and evaporation of the solvent with a yield of 8.9% (w/w). DMF was isolated from KPE and identified as described previously [12], and its purity was confirmed to be ≥98% by high-performance liquid chromatography.

### Animal Experiment

Five-week-old male C57BL/6J (wild-type) and C57BL/6J (*ob/ob*) mice were purchased from Japan SLC, Inc., (Shizuoka, Japan) and housed under conditions of 55 ± 5% humidity, a 12 h day/night cycle, and 25 ± 2°C. All mice were provided with a normal chow diet (Rodent Chow 38057; Purina Irradiated Lab., USA) and water *ad libitum* throughout the experiment. After a 3-week acclimatization period, wild-type mice were assigned to the WT group, and *ob/ob* mice were randomly divided into three groups with comparable average body weights. All outcome assessments were performed by investigators blinded to group allocation. The experimental groups were as follows: (1) WT group (control, *n* = 6); (2) *ob/ob* group (obesity-induced muscle atrophy model, *n* = 6); (3) DMFL group (*ob/ob* treated with low-dose DMF, 20 mg/kg/day, *n* = 6); and (4) DMFH group (*ob/ob* treated with high-dose DMF, 50 mg/kg/day, *n* = 6). Mice in the WT and *ob/ob* groups were given an equal volume of saline to that provided in the DMF-treated groups. Body weight and food intake were measured twice a week. After the mice were sacrificed, skeletal muscles including the gastrocnemius (GA), tibialis anterior (TA), extensor digitorum longus (EDL), and soleus (SOL) and white adipose tissues (WAT), consisting of subcutaneous (sWAT), perirenal (pWAT), and epididymal (eWAT) depots, were dissected, weighed, and immediately snap-frozen in liquid nitrogen before storage at -80°C. All the experimental protocols were reviewed and approved by the Institutional Animal Care and Use Committee (IACUC) of the Yonsei Laboratory Animal Research Center (Permit No.: IACUC-A-201610-416-04).

### Micro-Computed Tomography Imaging

Micro-computed tomography (Micro-CT) imaging was performed using an animal positron emission tomography/CT/single photon emission tomography (INVEON; Siemens, USA), and all scans were acquired and analyzed at the Pohang Center for Evaluation of Biomaterials (Pohang Technopark, Republic of Korea).

### Grip Strength Test

The grip strength of mice was evaluated using a Chatillon force measurement system (Columbus Instrument, USA) equipped with a pull bar. Fore/hindlimb and forelimb grip strengths were measured at the end of the oral administration period. The system has an electronic digital force gauge that determines the peak force. Each mouse was held by the tail until it released the pull bar. Five consecutive tests were performed on each mouse to obtain the peak value.

### Treadmill Test

Running endurance was assessed using an animal treadmill (LE8710MTS, Panlab, Spain). Mice were acclimated to the apparatus by running at 12 m/min on a 0° incline prior to testing. During the endurance test, the treadmill was initially set at 12 m/min with no incline, and the speed was increased by 3 m/min every 20 min. After 60 min, the incline was raised by 5° at 20 min intervals. A shock grid delivering 0.2 mA was used as a mild stimulus that did not cause physical harm. Exhaustion was defined as the time point at which a mouse failed to resume running after receiving the electrical stimulus for 10 s.

### Analysis of Blood Biochemical Parameters

Blood samples were collected from all mice by heart puncture and incubated at room temperature. Serum was prepared by centrifugation of the blood at 4000 rpm for 15 min, and stored at -80°C until analysis. Serum lipid profiles, including total triglycerides (TG), total cholesterol (TC), high-density lipoprotein cholesterol (HDL-C), and low-density lipoprotein cholesterol (LDL-C), as well as the hepatotoxicity marker alanine aminotransferase (ALT), were measured using an automated biochemical analyzer (Mindray, Nanshan, Shenzhen, China) according to the International Federation of Clinical Chemistry guidelines and the manufacturer’s instructions.

### Histological Analysis

GA muscle and eWAT fixed with 10% formalin solution were embedded in paraffin and stained with hematoxylin and eosin (H&E). The stained tissues were observed under an inverted microscope equipped with twin charge-coupled device cameras (Eclipse TE2000U, Nikon, Japan). The adipocyte size and the cross-sectional area (CSA) of the muscle fiber were quantified using ImageJ software (version 1.47; National Institutes of Health, USA).

### Reverse Transcription Polymerase Chain Reaction (RT-PCR)

Homogenized eWAT and SOL/GA muscles were analyzed by RT-PCR according to the previous study [12].

### Western Blot Assay

Homogenized eWAT and GA/SOL muscles were subjected to Western blot analysis following the procedures described in a previous study [16]. The primary antibodies against phospho-AMPK (p-AMPK, Thr172; #2531), AMPK (#2532), phospho-acetyl-CoA carboxylase (p-ACC, Ser79; #3661), ACC (#3662), peroxisome proliferator-activated receptor gamma (PPARγ, #2435), CCAAT/enhancer-binding protein alpha (C/EBPα, #2295), phospho-PI3K (p-PI3K, Tyr458/Tyr199; #4228), PI3K, phospho-Akt (p-Akt, Ser473; #4292), Akt (#9272), phospho-mammalian target of rapamycin (p-mTOR, Ser2448; #2971), mTOR (#2972), phospho-70-kDa ribosomal protein S6 kinase (p-p70S6K, Thr389; #9205), p70S6K (#9202), phospho-eukaryotic initiation factor 4E-binding protein 1 (p-4EBP1, Thr37/46; #2855), 4EBP1(#9452), phospho-forkhead box O3a (p-FoxO3a, Ser253; #9464), FoxO3a (#2497), uncoupling protein 2 (UCP2, #89326), and α-tubulin (#2144) were purchased from Cell Signaling Technology (USA) and used in a 1:1000 dilution. Sterol regulatory element-binding protein 1c (SREBP-1c, sc-365513), PPARγ coactivator 1-alpha (PGC-1α, sc-518025), UCP3 (sc-31387), were purchased from Santa Cruz Biotechnology (USA) and used in a 1:1000 dilution. Horseradish peroxidase-conjugated secondary antibodies (1:2500 dilution; Bethyl Laboratories, Inc., USA) were used to visualize the proteins on the membrane. The proteins were detected using an enhanced chemiluminescence detection solution (Amersham Biosciences, UK) and visualized with the image analysis system (NFEC-2025-08-307766).

### Molecular Docking

The X-ray crystal structure of PI3K (PDB ID: 8OW2) [17] and AMPK (PDB ID: 4CFF) [18] were obtained from the RCSB Protein Data Bank [19]. The 3D structure of DMF was retrieved from PubChem (ID: 88881). Protein preparation was conducted using AutoDockTools (ADT) 1.5.7, as previously described [20], and molecular docking was performed using AutoDock Vina within the PyRx platform [21]. The search grid was defined as an exhaustiveness value of 9. For PI3K (8OW2), the grid center was set to (0.000, 2.000, -3.000) with dimensions of 20 × 20 × 20 Å^3^. For AMPK (4CFF), the center was set to (-27.000, -7.000, 205.000) with dimensions of 20 × 20 × 26 Å^3^. Final protein-ligand interactions were visualized and analyzed using PyMOL 3.1.0 (Schrödinger, USA).

### Statistical Analysis

Statistical differences among groups were evaluated using one-way analysis of variance (ANOVA), followed by Tukey’s post hoc test when applicable. In cases requiring pairwise comparisons, unpaired *t*-tests were performed. All analyses were conducted using GraphPad Prism 10.0 (GraphPad Software, USA). Data are reported as the mean ± standard deviation (SD) and a *p*-value of <0.05 was considered indicative of statistical significance.

## Results

### DMF Improved Obesity-Related Metabolic Parameters in *ob/ob* Mice

**Fig. 1:**
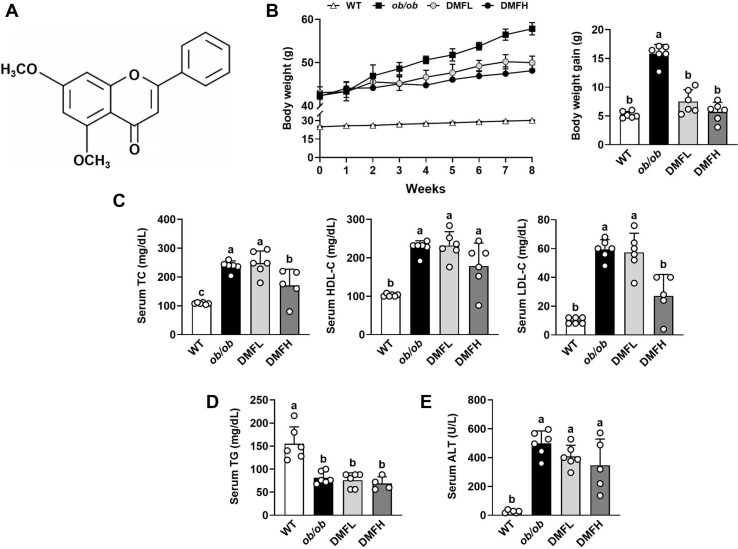
Effects of DMF on body weight regulation and serum lipid profiles in *ob/ob* mice. (**A**) Chemical structure of DMF. (**B**) Weekly body weight measurements during the 8-week treatment period and body weight gain at the end of the experiment. *n* = 6 per group. (**C**) Serum levels of total cholesterol (TC), high-density lipoprotein cholesterol (HDL-C), and low-density lipoprotein cholesterol (LDL-C). *n* = 6 per group. (**D**) Serum triglyceride (TG) concentrations. (**E**) Serum alanine aminotransferase (ALT) levels. *n* = 6 per group. Data are presented as mean?±?SD, and bars not sharing a common letter denote statistically significant differences between groups (*p* < 0.05).

To investigate the anti-obesity effects of DMF (Fig. 1A), *ob/ob* mice were orally administered DMF at 20 mg/kg/day (DMFL) or 50 mg/kg/day (DMFH) for 8 weeks. The *ob/ob* control group exhibited a progressive and significant increase in body weight throughout the 8-week period. In contrast, the administration of both low and high doses of DMF significantly suppressed this body weight gain compared to the untreated *ob/ob* control group (Fig. 1B). We next assessed serum lipid parameters and the hepatotoxicity marker ALT to determine whether DMF alleviated obesity-associated metabolic disturbances. The *ob/ob* control mice displayed severe hypercholesterolemia. Specifically, serum levels of TC, HDL-C, and LDL-C were significantly increased in the *ob/ob* group, and these increases were attenuated by high-dose DMF treatment (Fig. 1C). Serum TG levels were also significantly decreased in the *ob/ob* group; however, there was no significant difference observed following DMF treatment (Fig. 1D). Furthermore, the *ob/ob* group showed dramatically elevated serum ALT levels, an indicator of liver damage, but this increase was not significantly reversed by either DMF treatment (Fig. 1E). Notably, DMF-treated mice showed reduced body weight gain without changes in food intake (data not shown), implying a potential modulation of energy expenditure.

### DMF Reduced Fat Accumulation and Adipocyte Hypertrophy in *ob/ob* Mice

**Fig. 2:**
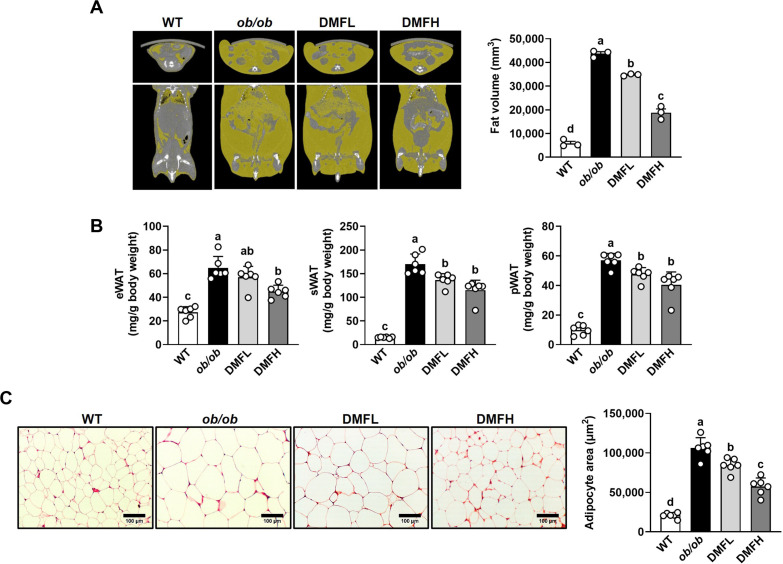
Effects of DMF on adiposity in *ob/ob* mice. (**A**) Abdominal fat volume was quantified using micro-CT imaging. *n* = 3 per group. (**B**) Weights of epididymal white adipose tissue (eWAT), subcutaneous white adipose tissue (sWAT), and perirenal white adipose tissue (pWAT). *n* = 6 per group. (**C**) Representative H&E-stained sections of eWAT and quantification of adipocyte area. *n* = 6 per group. Data are presented as mean?±?SD, and bars not sharing a common letter denote statistically significant differences between groups (*p* < 0.05).

To further confirm the anti-obesity effect of DMF, we analyzed fat accumulation using Micro-CT and measured adipose tissue weights. The Micro-CT analysis revealed that the total fat volume, which was dramatically increased in the *ob/ob* group, was significantly reduced by both low and high doses of DMF (Fig. 2A). Consistent with this, the normalized weights of eWAT, sWAT, and pWAT were all significantly elevated in the *ob/ob* group. This increase was significantly suppressed in both DMF-treated groups compared to the *ob/ob* control group (Fig. 2B). We also performed histological analysis of the eWAT. The *ob/ob* control mice exhibited severe adipocyte hypertrophy, characterized by an increase in adipocyte size (Fig. 2C). Both DMFL and DMFH treatments significantly suppressed this adipocyte hypertrophy, resulting in a markedly reduced average adipocyte area compared to the untreated *ob/ob* mice. Collectively, these results indicate that DMF treatment effectively attenuates obesity in *ob/ob* mice by inhibiting fat accumulation and reducing adipocyte hypertrophy.

### DMF Activated AMPK Signaling and Inhibited Adipogenesis and Lipogenesis in eWAT

**Fig. 3:**
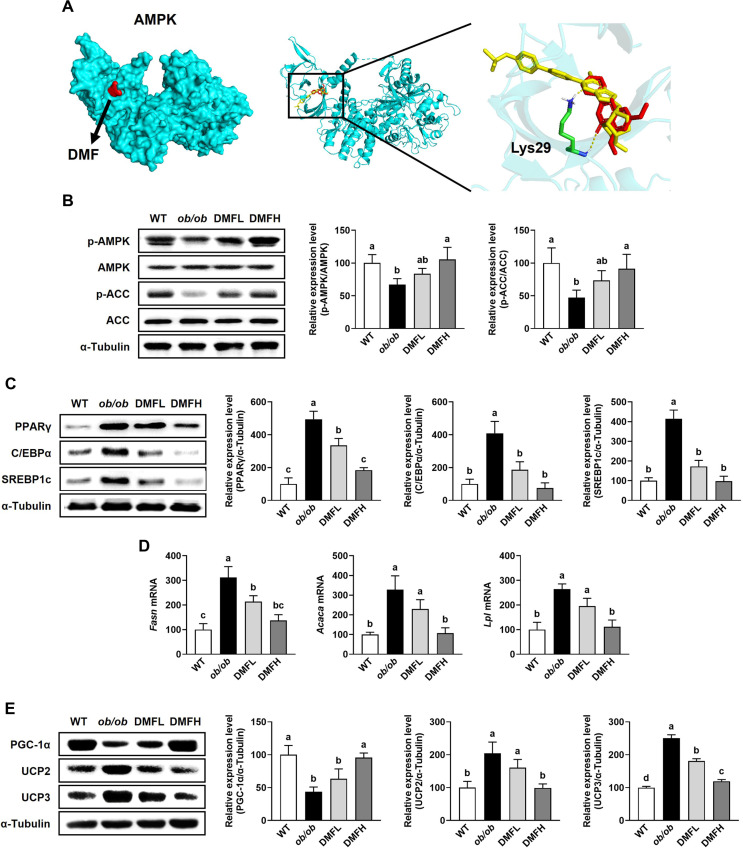
Effects of DMF on adipogenesis, lipogenesis, and thermogenesis via AMPK activation in eWAT of *ob/ob* mice. (**A**) Overview of DMF binding to the AMPK structure. Superimposed poses of DMF (red) and activator A-769662 (yellow), demonstrating similar binding orientations in the allosteric regulatory site. (**B**) Protein expression levels of phosphorylated and total AMPK and ACC were analyzed by Western blotting. *n* = 3 per group. (**C**) Protein expression levels of the adipogenic transcription factors, such as PPARγ, C/EBPα, and SREBP-1c, assessed via Western blotting. *n* = 3 per group. (**D**) The mRNA expression levels of lipogenic genes, including *Fasn*, *Acaca*, and *Lpl*, determined by RT-PCR. *n* = 3 per group. (**E**) Protein expression levels of thermogenesisrelated markers, including PGC-1α, UCP2, and UCP3, assessed via Western blotting. *n* = 3 per group. Data are presented as mean ± SD, and bars not sharing a common letter denote statistically significant differences between groups (*p* < 0.05).

To investigate the potential molecular pathways by which DMF suppresses adiposity, we first investigated whether DMF could interact with AMPK using *in silico* molecular docking simulation. The analysis suggested a structural plausibility for DMF binding stably within a key allosteric pocket of the AMPK protein (Fig. 3A). Notably, this binding site is consistent with the location utilized by other known direct AMPK activators, such as A-769662. The stability of this interaction is reinforced by specific hydrogen bonds formed between DMF and key amino acid residues in the pocket, including Lys 29. Consistent with this *in silico* prediction, we next assessed the *in vivo* activation of AMPK, a master regulator of energy metabolism. The levels of phosphorylated AMPK and its downstream target, phosphorylated ACC, were significantly reduced in the *ob/ob* compared to the WT group (Fig. 3B). Conversely, DMF administration, particularly at the high dose, significantly restored the phosphorylation of both AMPK and ACC, indicating a potent activation of the AMPK signaling pathway.

Since AMPK activation is known to inhibit adipogenesis [22], we next measured the protein expression of major adipogenic transcription factors. The protein levels of PPARγ, C/EBPα, and SREBP-1c were dramatically upregulated in the *ob/ob* group (Fig. 3C). Treatment with both DMFL and DMFH significantly suppressed the elevated expression of these transcription factors. Furthermore, we examined the mRNA expression of downstream lipogenic enzymes. Consistent with the reduction in transcription factors, the elevated mRNA levels of lipogenic genes, including fatty acid synthase (*Fasn*), acetyl-CoA carboxylase alpha (*Acaca*), and lipoprotein lipase (*Lpl*), in the *ob/ob* group were also significantly decreased by DMF treatment (Fig. 3D). Given the close link between AMPK activation and thermogenic regulation, we also assessed thermogenesis-related protein markers. DMF administration restored the reduced PGC-1α level in the *ob/ob* group while suppressing the elevated expression of UCP2 and UCP3 (Fig. 3E). Taken together, these data suggest that DMF attenuates adiposity and fat accumulation is associated with the activation of the AMPK signaling pathway, which in turn inhibits the expression of adipogenic and lipogenic factors while normalizing thermogenesis-related markers in adipose tissue.

### DMF Alleviated Obesity-Induced Muscle Atrophy in *ob/ob* Mice

**Fig. 4:**
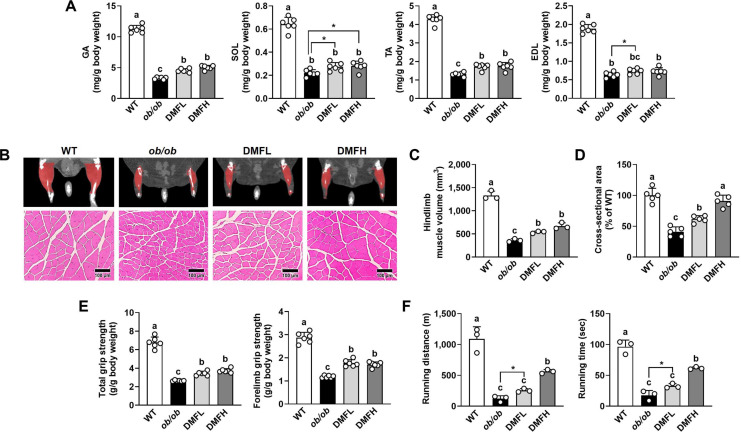
Effects of DMF on muscle mass and muscle function in *ob/ob* mice. (**A**) Weights of the gastrocnemius (GA), soleus (SOL), tibialis anterior (TA), and extensor digitorum longus (EDL) muscles. (**B**) Representative H&E-stained histological images of GA muscle sections. (**C**) Hindlimb muscle volume quantified using micro-CT imaging. *n* = 3 per group. (**D**) Quantification of myofiber cross-sectional area (CSA). *n* = 4 per group. (**E**) Forelimb and combined fore/hindlimb grip strength measured using a grip strength meter. *n* = 6 per group. (**F**) Running time and running distance assessed by treadmill performance testing. *n* = 3 per group. Data are presented as mean ± SD, and bars not sharing a common letter denote statistically significant differences between groups (*p* < 0.05).

A critical problem increasingly associated with obesity is the reduction of skeletal muscle mass, known as obesity-associated muscle loss [23]. Therefore, we next investigated whether DMF could also alleviate the muscle atrophy observed in *ob/ob* mice. To determine whether DMF could counteract the muscle loss associated with obesity, we assessed skeletal muscle mass. The *ob/ob* control mice exhibited significantly lower normalized muscle weights for the GA, SOL, TA, and EDL compared to the WT group, confirming a significant reduction in muscle mass associated with obesity (Fig. 4A). Treatment with both low and high doses of DMF significantly increased the normalized weights of all measured skeletal muscles compared to the untreated *ob/ob* controls.

This anti-atrophic effect was further confirmed by Micro-CT imaging and histological analysis. Three-dimensional reconstructions from Micro-CT scans showed the severe reduction in hindlimb muscle mass in *ob/ob* mice, which was restored by DMF treatment (Fig. 4B). Quantification of the hindlimb muscle volume demonstrated that the severe reduction observed in the *ob/ob* group was significantly reversed by both DMFL and DMFH administration (Fig. 4C). Consistent with the whole-muscle data, histological analysis revealed marked muscle fiber atrophy in the GA muscle of *ob/ob* mice. This was quantified by measuring the CSA, which was significantly and dose-dependently increased by both DMFL and DMFH treatments compared to the *ob/ob* controls (Fig. 4B and 4D).

To determine if the observed increase in muscle mass translated to functional improvements, we next assessed muscle strength and exercise endurance. Consistent with their obesity-induced muscle dysfunction, the *ob/ob* control mice exhibited significantly reduced grip strength compared to the WT group (Fig. 4E). Both DMFL and DMFH treatments significantly reversed this functional decline, restoring muscle strength to levels significantly higher than the untreated *ob/ob* controls. In addition to strength, exercise endurance was evaluated using a treadmill test. The *ob/ob* mice demonstrated severely impaired exercise capacity, with both running distance and running time being significantly lower than in the WT group (Fig. 4F). DMF administration significantly reversed this impairment. Collectively, these data demonstrate that the DMF-mediated increase in muscle mass translates to enhanced muscle function, improving both strength and endurance in *ob/ob* mice.

### DMF Activated the PI3K/Akt/mTOR Anabolic Signaling Pathway in GA Muscle

**Fig. 5:**
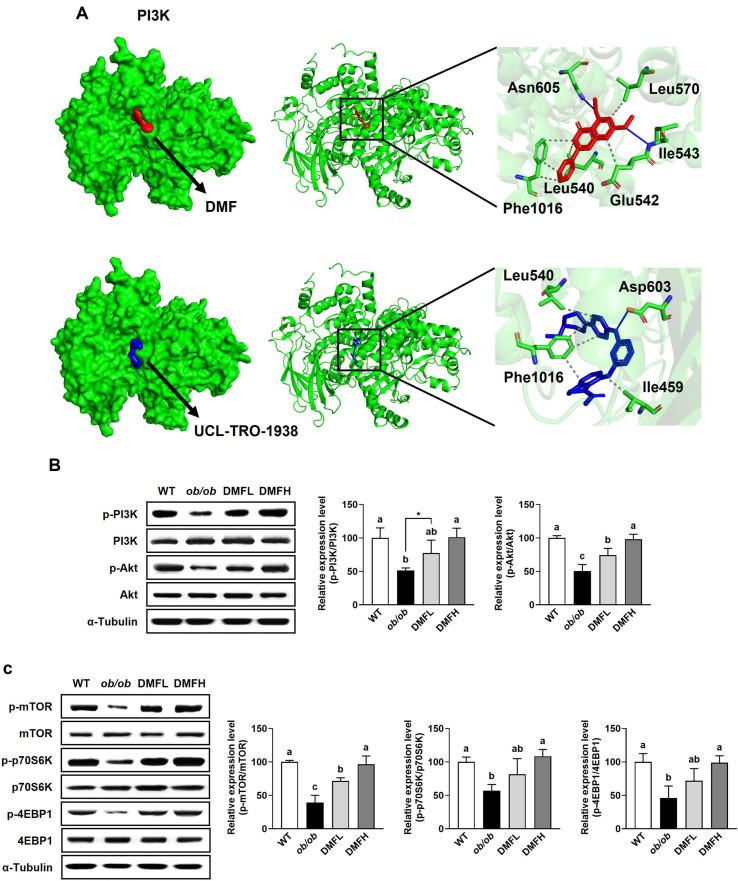
Effects of DMF on protein synthesis-related pathway in GA muscle of *ob/ob* mice. (**A**) Molecular docking simulation of DMF (red, top) and the activator UCL-TRO-1938 (blue, bottom) binding to the PI3K structure (green). (**B**) Protein expression levels of phosphorylated and total PI3K and Akt were analyzed by Western blotting. *n* = 3 per group. (**C**) Protein expression levels of phosphorylated and total mTOR, p70S6K and 4EBP1 were analyzed by Western blotting. *n* = 3 per group. Data are presented as mean ± SD, and bars not sharing a common letter denote statistically significant differences between groups (*p* < 0.05).

To investigate potential signaling pathways underlying the anti-atrophic effects of DMF, we first examined whether DMF could bind to PI3K using an *in silico* molecular docking analysis. The simulation predicted that DMF occupies the same binding pocket as the known activator, UCL-TRO-1938 (Fig. 5A). To validate this predicted activation, we next examined the PI3K/Akt/mTOR signaling pathway, a critical regulator of muscle protein synthesis, in GA muscle tissue. We found that the phosphorylation levels of PI3K and Akt were significantly suppressed in the *ob/ob* group compared to the WT group (Fig. 5B). Administration of both low and high doses of DMF significantly restored the phosphorylation of both PI3K and Akt. We next examined key downstream targets of this pathway (Fig. 5C). Consistent with the upstream findings, the phosphorylation of mTOR, p70S6K, and 4EBP1 was markedly reduced in the *ob/ob* group. Treatment with both DMFL and DMFH significantly reversed this suppression. These data suggest that the muscle-protective effects of DMF are associated with the activation of the PI3K/Akt/mTOR anabolic signaling cascade in the skeletal muscle of *ob/ob* mice.

### DMF Inhibited Muscle Atrophy Pathways and Restored Mitochondrial Biogenesis Markers

**Fig. 6:**
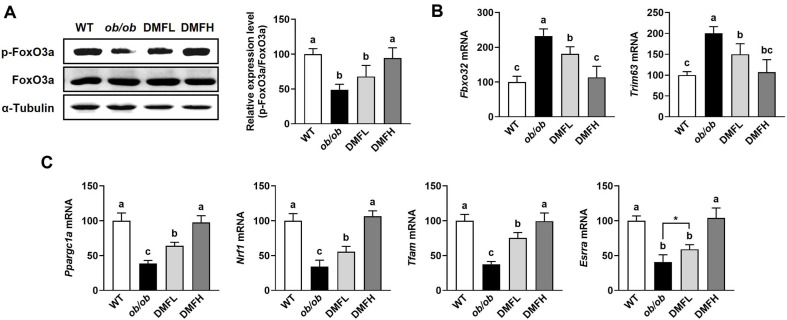
Effects of DMF on protein degradation and mitochondrial biogenesis–related markers in GA and SOL muscles of *ob/ob* mice. (**A**) Protein expression levels of phosphorylated and total FoxO3a analyzed by Western blotting. *n* = 3 per group. (**B**) The mRNA expression levels of muscle-specific E3 ubiquitin ligases, including *Fbxo32* (Atrogin-1) and *Trim63* (MuRF1) in GA muscle. *n* = 3 per group. (**C**) The mRNA expression levels of mitochondrial biogenesis– related genes, including *Ppargc1a*, *Nrf1*, *Tfam*, and *Esrra*, in SOL muscle. *n* = 3 per group. Data are presented as mean ± SD, and bars not sharing a common letter denote statistically significant differences between groups (*p* < 0.05).

To further confirm the anti-atrophic mechanism downstream of Akt, we examined the phosphorylation of FoxO3a. Akt activation leads to the inhibitory phosphorylation of FoxO3a, preventing its nuclear translocation [24]. The level of phosphorylated FoxO3a was significantly reduced in the *ob/ob* group compared to WT mice. DMF treatment, particularly at the high dose, significantly restored this phosphorylation, indicating the inhibition of FoxO3a activity (Fig. 6A). FoxO3a regulates the transcription of muscle-specific E3 ubiquitin ligases [24]. Consistent with the increased inhibitory phosphorylation of FoxO3a, the dramatically elevated mRNA expression of *Fbxo32* (muscle atrophy F-box protein 1, Atrogin-1 or MAFbx) and Trim63 (muscle RING-finger protein-1, MuRF1) observed in the *ob/ob* group was significantly and dose-dependently suppressed by DMF treatment (Fig. 6B).

Finally, given the improvements in exercise endurance, we assessed markers of mitochondrial biogenesis in SOL muscle. The mRNA expression levels of peroxisome proliferator-activated receptor-gamma coactivator 1-alpha (*Ppargc1a*), nuclear respiratory factor 1 (*Nrf1*), mitochondrial transcription factor A (*Tfam*), and estrogen-related receptor alpha (*Esrra*) were significantly decreased in the *ob/ob* group (Fig. 6C). DMF administration, using both low and high doses, significantly reversed this decline, restoring the expression of these key mitochondrial regulators.

## Discussion

There have been numerous efforts to identify natural products capable of counteracting obesity [25]. Among these, only a limited number have demonstrated concurrent muscle-hypertrophic effects [16, 26]. Nevertheless, no drug has been approved for obesity-induced muscle atrophy, emphasizing the need for agents that can both lower adiposity and maintain muscle mass. This study provides novel evidence of the dual function of DMF on both obesity and muscle atrophy in *ob/ob* mice. Collectively, our findings suggest that DMF is associated with the stimulation of skeletal muscle anabolism via the PI3K/Akt pathway and the suppression of adiposity through AMPK signaling.

Obesity is characterized by increased body weight caused by abnormal adipose tissue growth [27]. During the past decade, AMPK has been targeted as therapeutic approach for obesity treatment since it plays a pivotal role in energy metabolism [28, 29]. Activation of AMPK inhibits fatty acid synthesis via the inactivation of lipogenic enzymes such as ACC1, 3-hydroxy-3-methylglutaryl-coenzyme A reductase (HMGR), and FAS [28]. Moreover, activation of AMPK inhibits adipogenesis by downregulating PPARγ, C/EBPα, and SREBP-1c, which are highly expressed during adipocyte differentiation and regulate the expression of multiple adipogenic proteins such as LPL, glucose transport-4, and adipocyte fatty acid-binding protein [30-32]. Furthermore, AMPK enhances metabolic efficiency by promoting fatty acid oxidation and thermogenic activity through the upregulation of PGC-1α and uncoupling proteins [33]. In this study, DMF effectively decreased fat mass and volume and concurrently activated AMPK in eWAT. This *in vivo* activation is consistent with the structural plausibility suggested by our *in silico* docking analysis, which predicted that DMF binds to a key allosteric pocket of AMPK, a site consistent with other known activators such as A-769662. Consequently, this activation led to the decreased expression of the adipogenic transcription factors including PPARγ, C/EBPα, and SREBP-1c, and lipogenic enzymes, including *Fasn*, *Acaca*, and *Lpl*. UCP2 and UCP3 protein expression was lower in DMF-treated mice than in untreated *ob/ob* control mice. This reduction is likely attributable to the mitigation of adipose tissue dysfunction by DMF administration, as decreased lipid burden and improved mitochondrial homeostasis reduce the need for stress-driven UCP upregulation. Taken together, DMF effectively reduces obesity and downregulates adiposity via the activation of the AMPK signal cascade in fat tissue. Interestingly, DMF treatment altered body composition without affecting food intake. Although we did not directly measure energy expenditure or physical activity levels, a limitation of the current study, the simultaneous reduction in fat mass and increase in muscle mass suggest a potential shift in energy partitioning. In line with this, KPE, from which DMF is derived, has been reported to increase whole-body energy expenditure in healthy human subjects, potentially through activation of brown adipose tissue thermogenesis, suggesting that DMF may also influence basal metabolic rate and overall energy metabolism [34]. The activation of AMPK in adipose tissue and the PI3K/Akt pathway in muscle may collectively enhance metabolic efficiency, redirecting energy substrates toward muscle anabolism rather than adipose storage. Further metabolic profiling using indirect calorimetry will be necessary to confirm these physiological changes.

In previous studies, polymethoxylated flavonoids such as nobiletin and tangeretin, the major bioactive components in citrus fruits, have modulated lipid metabolism in cells and animals [35, 36]. Nobiletin upregulates lipolysis in mature adipocytes, and tangeretin downregulates lipid synthesis by inhibiting the activity of diacylglycerol acyltransferase (DGAT) [35]. In addition, hydroxylated polymethoxyflavones (HPMFs) suppress adipogenesis-related transcription factors in 3T3-L1 adipocytes, and attenuate obesity in a high-fat diet-induced mouse model [36]. Notably, other active components of KPE together with DMF, such as 5,7,4’-trimethoxyflavone and 3,5,7,3’,4’-pentamethoxyflavone, have also been reported to reduce adipocyte hypertrophy by enhancing the activity of lipolytic enzymes in mature 3T3-L1 adipocytes [11, 37]. Considering these previous results, several methoxylated flavones, including DMF, appear to share common anti-obesity properties. Building on this, our results further indicate that DMF provides broader metabolic benefits by simultaneously improving skeletal muscle function in vivo, suggesting a wider therapeutic potential than other methoxylated flavones that mainly target adipocyte metabolism. Although the structural basis underlying these differences remains unclear, elucidating how methoxy or hydroxyl substitutions influence the metabolic activity of flavones will be essential.

An increase in type II muscle mass leads to muscle strength enhancement [38]. DMF significantly increased the CSA of muscle fibers, which in turn contributed to the greater weights of type II-dominant muscles, including the GA and TA. The increase in GA and TA muscle weights resulted in an increase in grip strength. In contrast, an increase in type I muscle mass causes an improvement of exercise endurance [39]. In the DMF-treated groups, SOL muscle mass along with running endurance was significantly increased. Also, DMF upregulated the mRNA levels of mitochondrial biogenesis-related biomarkers such as *Ppargc1a*, *Nrf1*, *Tfam*, and *Esrra*, in SOL muscle. An increased PGC-1α expression in SOL muscle is known to improve exercise endurance as it is a key regulator of mitochondrial biogenesis [40]. Previous studies have reported that KPE increases the amount of mitochondrial DNA and glycogen *in vivo*, and promotes energy production by upregulating ATP production and AMPK in C2C12 myocytes [41, 42]. Therefore, DMF may have enhanced running endurance by improving the function and quality of type I fibers, not just through its hypertrophy. However, extensive investigation is required to clarify which molecular mechanism of DMF has a direct effect on endurance exercise in obese mice.

These anabolic effects in muscle appear to be mediated by the PI3K/Akt pathway, one of the most critical pathways involved in muscle protein anabolism by the mTOR signaling [43]. Activation of mTOR is another critical event in skeletal muscle growth due to the fact that it promotes certain mechanisms such as protein formation and mitochondrial biogenesis by increasing the expression of insulin-like growth factor-1 (IGF-1) and PGC-1α, respectively [16, 43]. Furthermore, Akt phosphorylates FoxO3a protein, which is a key regulatory factor in protein degradation, leading to its sequestration in the cytoplasm away from its target genes, such as Atrogin-1 and MuRF1 [44]. In this study, DMF was associated with changes consistent with activation of the PI3K/Akt pathway in GA muscle. Our molecular docking simulation showed DMF binding to a pocket adjacent to the ATP-binding site as an allosteric activator, thereby stabilizing the ATP-protein interaction through key hydrogen bonds. This ATP-stabilizing role would enhance the kinase activity of PI3K, leading to the observed pathway activation. Consequently, this activation led to muscle growth by activating the mTOR signaling and repressing FoxO3a. Because an increase in Akt activity in muscle reduces adiposity as a secondary consequence of muscle growth, increased Akt activity by DMF might have contributed to fat reduction [38]. Moreover, by simultaneously promoting mTOR-mediated protein synthesis and suppressing FoxO3a-driven protein degradation, DMF effectively improved the balance of protein turnover in muscle. This coordinated regulation of anabolic and catabolic pathways likely contributes to the attenuation of muscle atrophy observed in DMF-treated *ob/ob* mice.

Concomitantly, obesity causes a chronic state of inflammation in which accumulated adipocytes release pro-inflammatory cytokines such as interleukin (IL)-6 or tumor necrosis factor-alpha (TNF-α), thus stimulating protein degradation in skeletal muscle and disrupting muscle function [45]. Because of this, natural products which exhibit anti-inflammatory effects, such as curcumin and quercetin, have shown to protect against obesity-induced muscle inflammation and atrophy [46, 47]. Meanwhile, DMF has been reported to exhibit an anti-inflammatory effect [48-50]. Accordingly, DMF may have attenuated the breakdown of skeletal muscle by decreasing the levels of inflammatory cytokines. It should be noted that the *ob/ob* mouse model, while effective for studying extreme obesity, primarily reflects leptin deficiency rather than the complex etiology of clinical sarcopenia, which involves aging, disuse, and chronic inflammation. Therefore, while we observed significant improvements in muscle mass and function, it remains unclear whether these effects result from a direct pharmacological action of DMF on muscle tissue or are secondary to the overall improvement in systemic metabolism and reduced lipid burden. Future studies utilizing primary myocytes or specific atrophy models are warranted to decouple these effects.

There are two important issues of DMF before it is investigated in humans: its pharmacokinetics and safety. It was reported that DMF quickly approached its maximal concentration within 2 hr after oral administration of KPE and was detected in various organs. However, KPE showed low oral bioavailability since the methoxyflavones were found in the feces as demethylated metabolites [51]. From these results, not only DMF but also its metabolites may have attenuated obesity and increased muscle anabolism in the present study. Further investigation is needed to determine how its metabolites work in adipose tissue and skeletal muscle. Another issue is its safety. Numerous studies have proven that KPE does not cause any pharmacotoxic signs nor abnormal alterations in both animals and humans [52, 53]. Nevertheless, DMF as a single compound has not been studied for its safety yet. Collectively, investigations into increasing its oral bioavailability and comprehensive toxicity studies need to be performed before its pharmaceutical effect is investigated in humans.

## Conclusion

In summary, DMF demonstrated a potent dual function in *ob/ob* mice, significantly reducing body fat while simultaneously increasing skeletal muscle mass and function. These effects were associated with tissue-specific mechanisms: AMPK activation in epididymal fat, which correlates with suppressed adipogenesis and lipogenesis, and PI3K/Akt pathway upregulation in skeletal muscle, which is linked to stimulated mTOR signaling and inhibited atrophic factors. Furthermore, DMF enhanced the expression of mitochondrial biogenesis-related markers in muscle. These pre-clinical data suggest that DMF is a promising functional candidate to attenuate obesity and obesity-related muscle dysfunction.
